# Longterm-habituation of the startle response in mice is stimulus modality, but not context specific

**DOI:** 10.3389/fnint.2013.00103

**Published:** 2014-01-09

**Authors:** Peter K. D. Pilz, Stephan W. Arnold, Anja T. Rischawy, Claudia F. Plappert

**Affiliations:** Animal Physiology, Institute of Neurobiology, University of TuebingenTuebingen, Germany

**Keywords:** long-term habituation, acoustic startle, tactile startle, context, generalization, specificity, auditory pathways

## Abstract

In mice, the specificity of longterm-habituation (LTH) of startle was tested in two experiments. In two strains of mice (C57Bl/6 and C3H) there was pronounced LTH over 10 days of acoustic stimulation in two different contexts of startle measurement. (We found LTH to be greater after stimulation with 14 kHz sine stimuli compared to noise or tactile stimuli). A change of context showed LTH to be independent of context, i.e., startle LTH in mice is a non-associative learning process. In the second experiment, 9 days of acoustic or tactile stimulation were given to C57B/6 mice. Both stimulus modalities produced LTH. When on the 10th day stimuli of the other modality were given, in both cases the long term habituated group showed no lower startle amplitude than a non-stimulated control group. This indicates LTH is stimulus-modality specific. Altogether, our results show that in mice—very similar to rats—LTH of startle is stimulus modality, but not context specific. In addition we found two indications that the LTH action site is on the sensory branch of the startle circuit.

## Introduction

Habituation is a sensory filtering process which decreases responses to repetitive stimuli. It is described in a well cited and surprisingly current review by Thompson and Spencer (1966), who characterize habituation by nine points. This characterization was updated 2009 (Rankin et al., 2009), and only one point was to be added: long-term habituation (LTH). Generally, two different forms of habituation are distinguished: firstly, short-term habituation (STH), which is normally referred to as simply “habituation” and which describes response decreases within one session, with inter-stimulus intervals mostly in the range of seconds or few minutes. Secondly, LTH, describing response decreases between sessions, most often over days (Leaton, 1976). Rankin et al. added LTH as a tenth point to the general habituation concept because this type of learning needs its own neuronal basis, differing from that of STH. LTH in mammals was mainly studied for suppression behaviors and for the acoustic startle response (ASR) (e.g. Leaton, 1976; Jordan et al., 2000). The dependence of LTH on e.g. contextual cues, which is one of the objectives of this study, sometimes differed between response systems studying suppressive behavior or startle (Jordan et al., 2000). Because LTH of the ASR is best understood, both behaviorally and concerning its neural circuitry (e.g. Groves et al., 1974; Leaton, 1976; Jordan and Leaton, 1982, 1983; Leaton and Supple, 1986; Jordan, 1989; Pilz and Leaton, 1999; Jordan et al., 2000), we wanted to address four questions concerning startle LTH in mice:

Firstly, the influence of context on LTH in mice was tested. If context cues are important for LTH in mice, this would point to an associative learning process. On the other hand, if LTH is independent of context, learning should be non-associative (Marlin and Miller, 1981; Jordan et al., 2000). Secondly, long-term changes of STH were studied. This influence of LTH has not been thoroughly examined. Mice are probably well suited for such an interaction study since in this species LTH and STH are slower, i.e., they need more stimuli for a similar decrease, than in rats, perhaps enabling us to describe the interaction better. We wanted to know whether STH remains constant during LTH, or whether it is influenced by LTH. The third goal was to test for stimulus modality specificity of LTH. Jordan and Poore (1998) found no specificity for stimulus frequency of LTH of the ASR; in the same study they found LTH of lick suppression to be frequency dependent. Startle LTH generalized from the training frequency to test stimuli with differing frequencies. Jordan and Poore argue that specificity indeed is one of the original nine characteristics of Thompson and Spencer (1966) for habituation, but that LTH of ASR nevertheless is well suited as paradigm to study habituation. Here we wanted to know whether LTH is modality specific (in more detail than done by Jordan and Leaton, 1982, see Introduction to Experiment 2), in which case at least a part of this original point of Thompson and Spencer would be confirmed.

Our fourth point was to interpret the results together with known facts of the neural startle pathway. This pathway consists of a sensory input branch, a sensory motor interface (giant neurons in the pontine reticular formation) and a motor output branch (Koch, 1999, for details see below). Two of the above results should indicate the action of LTH on this pathway. Recently it was shown that STH is located in the sensory input branch of the startle pathway (Pilz et al., 2004; Vogel and Wagner, 2005). If LTH decreases STH, this may indicate that the sensory input branch is where LTH modulates startle. In addition, it was also shown that the sensory input branches are modality specific, i.e., one input provides selectively auditory information to the sensory motor interface while a different input provides haptic information (Li and Yeomans, 1999; Scott et al., 1999; Simons-Weidenmaier et al., 2006). Thus, if LTH is stimulus modality specific, this would also indicate that the sensory input branch is the action site of LTH.

Two of these questions have already been addressed in rats, where it was shown that context has no influence on LTH learning of startle (Marlin and Miller, 1981; Jordan et al., 2000), while LTH of suppression behaviors is context dependent (Jordan et al., 2000). Since this type of experiment has, to our knowledge, never been repeated in a different species, we wanted to learn more about startle LTH in mice, which are currently quantitatively more important in behavioral research, and which sometimes differ from rats (Frick et al., 2000; Cressant et al., 2007; Snyder et al., 2009; Stranahan, 2011). The modality specificity has been partially addressed by Jordan and Leaton (1982). However, there are several reasons why we should do this experiment again with an experiment designed to answer only this question (see below, Introduction to Experiment 2). Furthermore, because we used three different stimuli to elicit LTH, we managed to find a stimulus that is probably best suited to elicit LTH in mice.

## Experiment 1: context specificity of LTH

One question of the first experiment was whether mice transfer LTH from one context to another. Therefore, in the following we trained mice for LTH to acoustic stimuli in one particular context, and then we tested their reaction to the same stimuli in a different context. We adopted two strains of mice, which possibly differ in their ability to learn contextual cues. When researching the learning of the prepulse inhibition paradigm, Plappert et al. (2006) found differences in amount and velocity of learning between C3H and Bl6 mice strains. These differences were believed to be due to different learning velocities, with Bl6 mice being slower in their ability to learn contextual cues. Indeed, a test of contextual influences partially confirmed this interpretation (Plappert et al., 2006). Thus, we expected either that context cues are unimportant for LTH in mice, similar to rats, or that contextual learning influences LTH. In the latter case, we expected a strain-dependent difference of this influence.

In contrast to the majority of studies about LTH in rats, we administered 100 stimuli per day. This was done in order to study not only LTH to the first responses of each day, but also STH and its change over days.

In addition, we show the results of a pretest, where we did not find reliable LTH to noise stimuli. This is in contrast to the effect of sine stimuli used in the main experiment, which consistently induced LTH. We consider this influence of stimulus quality to be interesting since—to our knowledge—there are no previous publications on the influence of stimulus quality on LTH.

### Methods

#### Subjects

We obtained 48 naïve female C57Bl/6J (“Bl6”) mice and 36 naïve female C3H/HeN (“C3H”) mice from Harlan Laboratories; 1 C3H was excluded because it did not startle on day one, resulting in 35 C3H. Twenty-four Bl6 mice were measured in a pretest, while the other mice (24 Bl6 plus 36 C3H mice) were measured in Experiment 1. Both strains were 6 weeks old at the beginning of the experiments. The mice were housed in groups of 3 to 4 in standard Macrolon cages containing nesting material under a 12-h light–dark schedule (lights on at 6 am) and received food and tap water *ad libitum*. The cages were in an air-conditioned room with the temperature set at 24°C, ±1°C and the humidity held at 60%, ±5%. The mice were adapted to the colony room for 14 days before testing began. Testing took place during the light period. Experiments were approved by the Regional Council of Tuebingen (ZP4/04).

#### Apparatus

Startle responses were measured inside a sound attenuated chamber by a movement sensitive piezo accelerometer platform (Startle-Messsystem, Universitaet Tuebingen, Germany) in one of two different contexts (see below). Movement-induced voltage changes were amplified and filtered (Low-Pass: 150 Hz; Piezo-Amp System, Universitaet Tuebingen, Germany) and then digitized with 1 kHz (DAP1200e in a standard personal computer; Microstar, Bellevue, WA). Startle amplitude was calculated as the difference between peak-to-peak voltage during a time window of 50 ms after stimulus onset and peak-to-peak voltage in the 50-ms time window before stimulus onset.

Stimuli and a continuous 45 dB broadband background noise were produced by a digital signal processing controlled system (Elf-Board with Siggen Software; Medav, Uttenreuth, Germany), amplified and emitted by a loudspeaker (Visaton HTM 5.6, Haan, Germany) inside the sound-absorbing chamber. Stimuli for all experiments had an intertrial interval of 15 s.

***Rectangular context 1.*** Mice were placed in a rectangular wire mesh test cage (5 × 8.5 × 5.5 cm) with an aluminum floor. The walls of the soundproof chamber (inside measure: 70 × 50 × 40 cm) were covered with dark yellow structured sound absorbing acoustic foam rubber. The chamber was illuminated by a white 5 W cold light bulb. The loudspeaker was located at the side of the test cage.

***Triangular context 2.*** Mice were put in triangular test cages with high Plexiglas walls, each wall 11 cm long (height: 30 cm) inside sound absorbing chambers of 45 × 55 × 65 cm. The walls of the sound absorbing chamber were covered with bright gray structured sound absorbing foam and vertical bright yellow stripes, illuminated by a white 5 W cold light bulb covered partially (in the direction of the mouse) by a clear bright red plastic sheet. [Since, according to Lall et al. (2010), mice cannot distinguish dark red, illumination was perhaps merely diminished]. The floor of the cage consisted of stainless steel bars (distance 0.7 cm). Below the bars there was a bin with filter paper strips covered with 10% anise oil (freshened before each experiment). The loudspeaker was situated above the test cage.

The two contexts differed with regard to size and geometry of test cage, floor of test cage, structure of test cage walls, color or brightness of illumination, different structures on the walls of the superstructure, odor, direction of sound stimulation, and, probably due to the sound reflecting walls of the triangular context, sensation of the continuous background noise. The triangular context was in a different room. Transportation of the mice (in the test cages) was longer with respect to time and length (3 doors, 30 m), compared to the other context (1 door, 5 m).

#### Procedure

Mice were startled on 10 consecutive days in one context. On the following 2 days they were then measured in the same or in a different context. Half of the mice of each strain were startled in the rectangular context in the first 10 days, while the other half was startled in the triangular context. Each half of this half was tested on day 11 in the same, and on day 12 in the different context. The other half was tested on day 11 in the different, on day 12 in the same context.

Each day the mice were adapted for 5 min to the context inside the sound absorbing chambers: they were exposed to the same background noise as during the following stimulation period. They were then exposed to 100 startle stimuli with an interval of 15 s. Startle stimulus consisted of 20 ms white noise 105 dB SPL in the pretest. In experiment 1 it was a 20 ms 14 kHz sine stimulus including 0.4 ms rise and fall times; here the SPL was 105 dB for the Bl6 mice and 100 dB for the C3H mice.

#### Statistical analysis

The startle responses were averaged for each day and mouse. Parametric statistics were then calculated with these averages. They were again averaged per strain over mice for days 1–10. Test days 11/12: ASR was averaged for the condition “same” = same context as LTH on day 1–10, and “different” = context differing from LTH context. Statistical analysis was done with JMP (SAS Institute, V. 10). LTH was tested over time by a repeated measures ANOVA on these response values. The Greenhouse-Geisser correction was used because Mauchly'sphericity test was significant (*p* < 0.05). LTH was tested on days 11/12 using dependent *t*-tests. In the case of multiple comparisons with day 1 (but not when comparing same context with different context), the *p*-value was Bonferroni corrected (factor 3).

In addition, for the LTH measures for each mouse and day, a relative value (percent of response of day 1) was calculated and the statistics repeated on these percentages. The test results for these relative measures were significant where statistics on absolute values were significant, and vice versa they were not significant for the cases where the tests on absolute measures were non-significant; therefore, these test results based on relative values are not reported separately.

STH was calculated as difference between mean first 10 and mean last 40 responses divided by the mean of all responses on the respective day of each mouse. These ratios were averaged over mice (per day), and a linear regression was calculated to test of changes of STH over days.

### Results

#### Pretest: no LTH with noise stimuli

With noise stimuli as startle stimuli, no significant LTH of the ASR could be observed (data not shown). The average ASR of days 9 + 10 was 96% of the ASR of day 1. This change was not significant [dependent *t*_(23)_ = 0.45, *p* = 0.66].

Since Plappert and Pilz (2005) could show reliable LTH with 14 kHz sine stimuli, this latter stimulus was used in the subsequent experiments. As can be seen below, tonal stimuli reliably induced LTH. Therefore, this pretest shows that acoustic stimulus quality influences LTH.

#### LTH is not context specific

**Figure 1:**
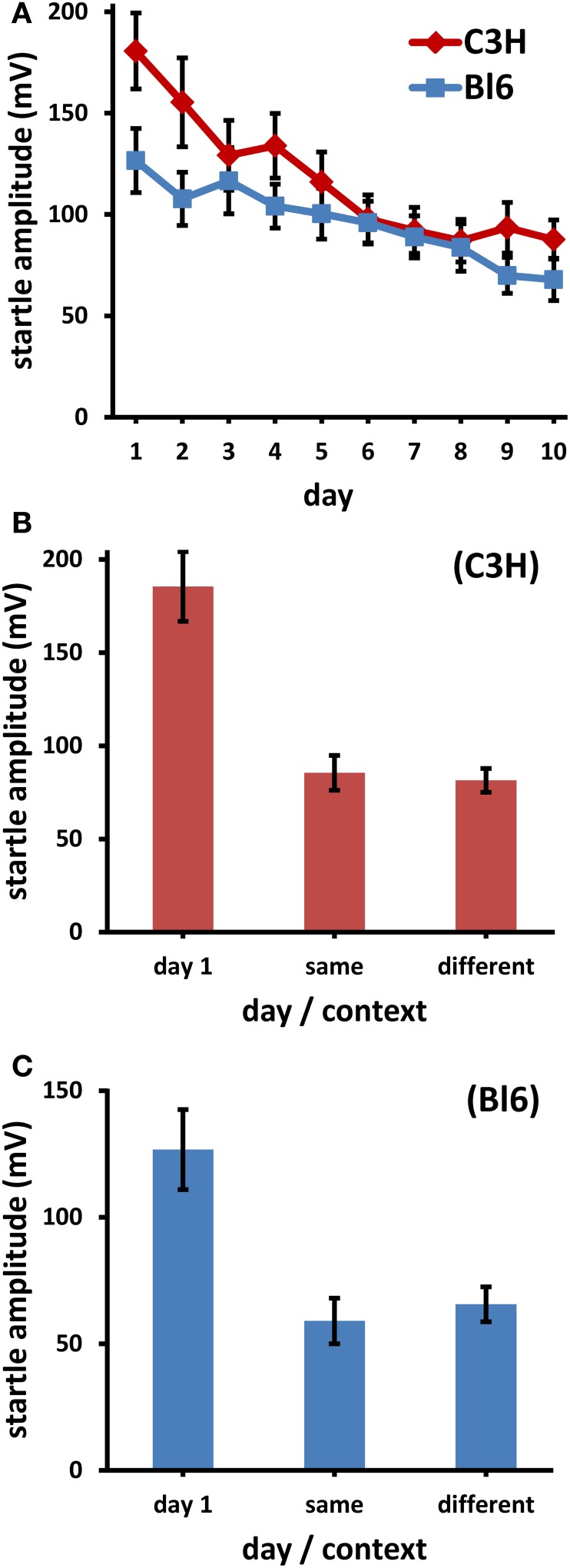
**(A)** Longterm habituation (LTH) to 14 kHz stimuli in two strains of mice (Bl6: C57Bl/6J, C3H: C3H/HeN) over 10 days. Each day comprises the average startle amplitude to 100 stimuli; the decrease was significant in both strains (*p* < 0.001). **(B)** C3H, **(C)** Bl6: average startle amplitude on day 1 (same as day 1 in **A**), and on day 11/12 in the same context or on day 11/12 in the different context. The decrease from day 1 to day 11/12 was significant (*p* < 0.01), the differences between contexts were not (*p* > 0.5). (Bars: s.e.m.; Bl6: *n* = 24, C3H: *n* = 35).

In Experiment 1, both strains showed reliable LTH (Figure 1A). In Bl6, the average response decreased on day 10 to 53.7% of day 1, in C3H to 48.5%. This decrease was highly significant [Bl6: *F*_(4.2, 78.9)_ = 6.82, *p* < 0.0001; C3H: *F*_(3.9, 137)_ = 13.0, *p* < 0.0001].

Half of the mice were tested on day 11 in the different context, the other half in the same context as that of days 1–10. On day 12 all mice were moved to the context different from day 11. The average ASR on the test days (11/12) was the same, regardless of whether it was measured in the same or in the different context (Figure 1B, C3H: ASR in the different context was 97.3% of ASR in same context on day 11/12. Figure 1C, Bl6: 108.8%). These small differences were insignificant (both strains: dependent *t* < 0.6, *p* > 0.5). Again, there was proof of significant LTH since in all cases the ASR was lower on days 11/12 than on day 1, irrespective of the context (dependent *t* ≥ 3.42, Bonferroni corrected *p* ≤ 0.0074).

#### Change of short-term habituation over days

**Figure 2:**
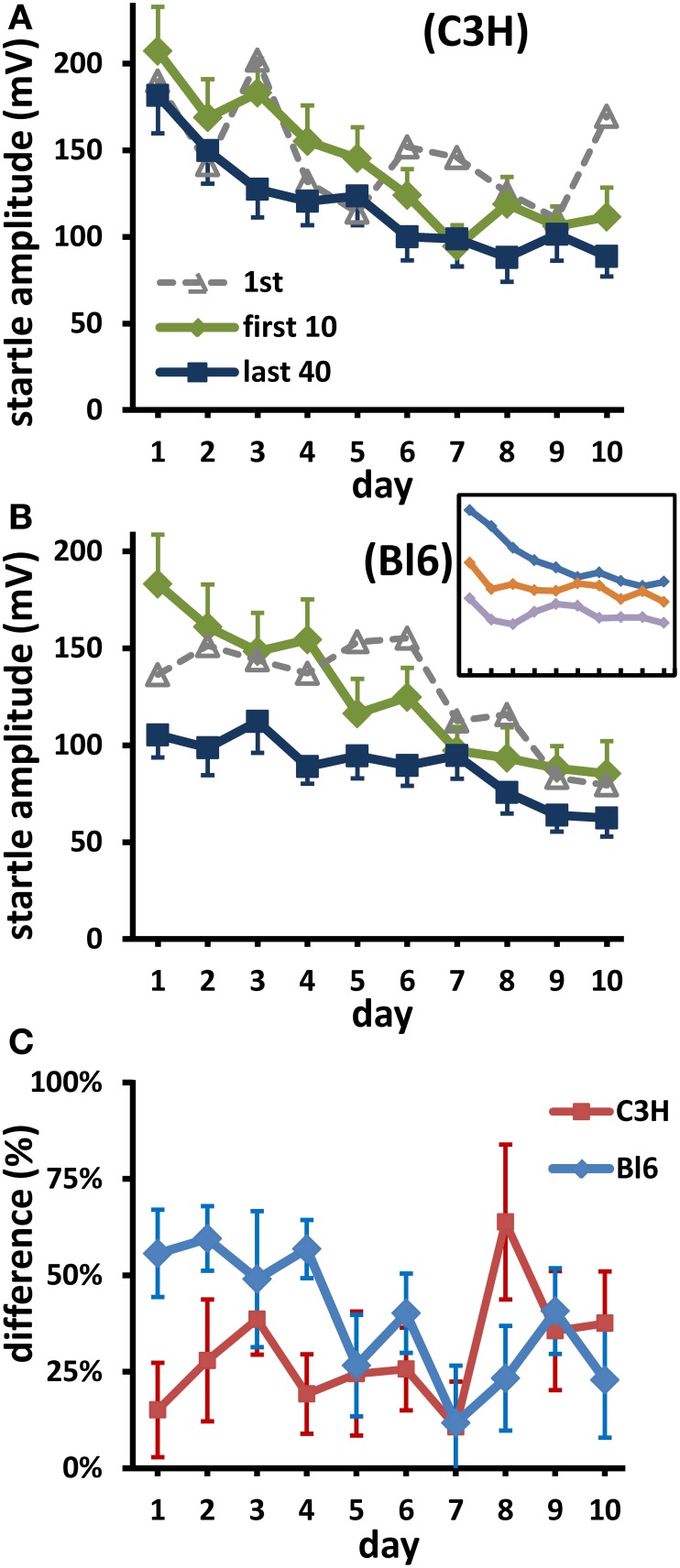
**Long-term habituation of short-term changes**. **(A)** (C3H), **(B)** (Bl6): Green lines are averages over the first 10 responses of each day; blue is the mean of the last 40 responses. For comparison, mean first responses are also shown (dotted lines). Inset in B (Bl6): short term habituation on day 1, 6, and 10 (from top to bottom; blocks of 10 responses). Bars: s.e.m.; for clarity, SEMs of 1st responses are not shown; they were always larger than SEMs of first 10 responses (mean factor: 1.87 in C3H, 1.48 in Bl6). **(C)** Short-term habituation (STH) on each day shown as mean percent change between mean first 10 and mean last 40 responses. The decrease of these differences over days was significant in Bl6 (*p* < 0.02), but not in C3H (*p* > 0.2).

With one small exception, at the end of each daily experiment, the mice startled less than at the beginning. Thus, the curves representing the last 40 responses are consistently below the curves of the first 10 responses (Figures 2A,B). In Bl6 mice this difference (representing absolute STH) during each measuring session decelerated over the course of 10 days (insert in Figure 2B). Relative STH (i.e., the percent change) also decreased over days; this decrease was significant in Bl6 mice (Figure 2C regression of percentages: *r*_(8)_ = −0.74, *p* = 0.014). In contrast, in C3H mice the responses at the beginning and the end of each day decreased in a similar manner during LTH (Figure 2A); the relative difference between both measures (Figure 2C) did not change significantly [*r*_(8)_ = 0.43, *p* = 0.21]. Thus, in Bl6 the amount of STH decreased over days. This was not the case in C3H, where STH was small from the beginning.

### Discussion

#### Pretest: no LTH with noise stimuli

We cannot explain why the mice showed no reliable LTH in the pretest with noise stimuli. Our data with an “LTH” to 96 %, i.e., only 4% decrease, coincide with Azzopardi et al. (2013), who also found only an LTH to 92% using a noise stimulus in the same strain of mice; Typlt et al. (2013) even observed a startle increase over days with noise stimulation. The results in the next experiment shown below with much higher LTH with sine stimuli confirm our own data in previous publications with a reliable LTH using this tonal stimulus (14 kHz, i.e., frequency of best hearing in mice: (Plappert and Pilz, 2005); 10 kHz, i.e., frequency of best hearing in rats: Pilz and Leaton, 1999). In contrast to these findings, Schmid et al. (2011) found a reliable LTH (about 50%) with noise stimuli in hybrid mice with a mixed C57Bl/6 × 129S6 genetic background. In a shorter test (over 5 days), we found no LTH in C3H mice to noise stimuli; however, we also found reliable LTH in C3H when tested over a longer period with 250 daily noise stimuli (unpublished data). Thus, while noise stimuli often result in no or almost no LTH, obviously a 14 kHz stimulus is a good stimulus to elicit LTH in mice.

We can only hypothesize why noise stimuli produce less LTH. One reason could be that noise stimuli are not as constant as tonal stimuli (neither in amplitude nor in frequency), and thus perhaps more difficult to learn. However, since Jordan and Poore (1998) showed no influence of acoustic stimulus frequency on LTH, this explanation is not convincing. Another reason might be that noise stimuli elicit long-term sensitization, thereby counteracting LTH, a process shown by Borszcz et al. (1989). This type of effect was reliably found in mice by Typlt et al. (2013). If this is the case, we still cannot explain why a noise stimulus should produce long-term sensitization, and a tonal stimulus should not.

To summarize the unpredicted stimulus dependency of LTH: in the following experiments and in future experiments we use 14 kHz stimuli to reliably elicit LTH in mice.

#### Main experiment: long-term habituation is not context specific

Both strains of mice transferred the LTH from one context to the other context (Figures 1B,C). The average startle responses were almost identical in the two different contexts. Therefore, we must conclude that context plays no role for LTH in mice.

This outcome confirms the findings of Marlin and Miller (1981) and Jordan et al. (2000) that LTH is not context specific in rats. Since we have now demonstrated that the same is true for mice, we believe we can apply this notion to rodents in general. In this group of animals it is obvious that startle LTH does not depend on association with context cues, confirming that LTH is a non-associative learning process.

Jordan et al. (2000) showed that this non-associative aspect of LTH is only true for startle LTH, and not for other long term habituation mechanisms for other paradigms. They discuss associative vs. non-associative models for LTH. Because they drew paradigm dependent conclusions, we refer the reader to this publication for discussion of the non-associative vs. associative nature of LTH in general. Their use of diverse response systems also offered the advantage of controlling the effect of background cue differences: tests of lick suppression, parallel to ASR testing, showed that the rats were able to differentiate between the contexts when this measure was evaluated.

It could be argued that in our study the mice did not learn the context cues available (Marlin and Miller, 1981). However, since we changed several cues in several sensory modalities simultaneously, for the purpose of startle testing in the lab, there is no measurable transfer of contextual learning between contexts.

#### Influence of long-term on short-term habituation

While in C3H mice the STH was small from the beginning, in Bl6 it was initially large and decreased over days. This leads to a smaller LTH in Bl6 compared to C3H, when all responses are used to characterize the daily ASR (Figure 1A). If only the first 10 responses are considered, LTH is much more similar between strains (compare “first 10” curves in Figures 2A,B). Since there was a reliable decrease of STH over days in Bl6, LTH interacted with this learning process. For the purpose of this discussion we evaluated data of another experiment in our lab, which did not strive to measure habituation and used more than 200 daily stimuli. In C3H we also found a significant STH on day 1; in this case STH also decreased significantly over days, paralleling the course of LTH (unpublished data).

We want to speculate about where LTH and STH interact in the startle pathway. Until now, all studies have suggested that the neuronal structure mediating LTH is an extrinsic pathway which modulates the startle pathway. Lesions to the mesencephalic reticular formation (MRF) (Jordan and Leaton, 1982; Jordan, 1989) or vermis of the cerebellum (Leaton and Supple, 1986, 1991; Lopiano et al., 1990) attenuate or eliminate LTH. Lesions of the inferior colliculus did not alter LTH (Jordan and Leaton, 1983), as did complete decerebration at the level of the mesencephalon (Leaton et al., 1985). Recently the involvement of the cerebellum in LTH has been confirmed in humans (Timmann et al., 1998; Maschke et al., 2000; Pissiota et al., 2002; Frings et al., 2006). Lesion of the MRF eliminates the long-term decrease of startle not only before, but also after LTH training (Jordan, 1989). Therefore, LTH is not a morphological change of e.g. synapses within the startle pathway, but LTH must be an extrinsic chronic modulation acting on this pathway. It must be chronic, since even fast EMG components of startle are decreased when they experience LTH [Poore and Jordan (1992); Jordan et al. (1993): cited in Jordan and Poore (1998)]. Thus, LTH chronically modulates the startle pathway at an unknown action site.

The auditory input from ear and acoustic nerve is common for auditory startle pathway, hearing and prepulse inhibition circuit (Plappert and Pilz, 2013). The neurons of the acoustic nerve are primary sensory neurons of the auditory startle circuit, followed by startle pathway specific secondary sensory neurons (Koch et al., 1992; Lingenhöhl and Friauf, 1992; Lee et al., 1996). The latter project onto giant neurons in the PnC (Koch et al., 1992; Lingenhöhl and Friauf, 1992), which are the sensory motor interface of startle. The motor output part begins as direct or indirect efferences of the giant neurons to motoneurons (Davis et al., 1982; Lingenhöhl and Friauf, 1994; Yeomans and Frankland, 1996). We believe we can exclude LTH acting at the level of the ear or acoustic nerve based on two findings: first, hearing is constant at this age of mice and at the frequency range used (Ehret, 1974); second, if processing up to the primary auditory neurons would be depressed by LTH, prepulse inhibition to acoustic prepulses should decrease during LTH, which is not the case (Plappert and Pilz, 2005). We also believe, however with much less confidence, that we can exclude LTH acting on the motor branch of the startle circuit. Due to the constant extrinsic inhibition by LTH, we would expect the decrease at the end of a day to be similar to that at the beginning, i.e., the percent STH should remain roughly constant during LTH (which is not the case). Thus, we are left with the startle specific secondary neurons or the giant PnC neurons. Several mechanisms may be at work here. The first possibility is the direct interaction of LTH with STH. Since STH of startle is a process situated in the sensory input branch (Pilz et al., 2004; Simons-Weidenmaier et al., 2006; Schmid et al., 2011), this would also place LTH in this branch. A second possibility would be that LTH acts selectively on neurons or nuclei processing higher auditory input. Meloni and Davis (1998), for example, have shown that the dorsal cochlear nucleus (DCN) contributes to a high intensity component of the acoustic startle reflex. LTH acting on this nucleus would result in the patterns we observed: LTH and a decrease of STH since the dynamic range of startle is restricted to a lower input range. Another possibility is that LTH selectively inhibits large caliber PnC-neurons, which would also result in a similar pattern. More complicated schemes may also come to mind, e.g. a decrease of STH over days independent of LTH, in which case no part of the pathway can be excluded as an action site of LTH. However, since only the first two mechanisms discussed here are based on current literature (and are more simple in the sense of Ockham's razor), we believe that the LTH action probably takes place on the sensory side of the startle pathway.

## Experiment 2: stimulus modality specificity of LTH

In this experiment we wanted to know whether LTH is specific for the modality of the learned stimulus. Stimulus specificity and stimulus generalization are important characteristics of habituation (Thompson and Spencer, 1966; Rankin et al., 2009). STH of startle is stimulus modality specific. If startle is short term habituated to one modality (tactile or acoustic), there is no generalization of this habituation to a different modality (Pilz et al., 2004; Vogel and Wagner, 2005). There are no such thorough analyses for LTH. In rats, daily acoustic stimuli elicited more LTH in controls than in rats with a lesion of the mesencephalic reticular formation (MRF; Jordan and Leaton, 1982). This difference between lesioned and control rats was not extended to tactile stimulation. While this suggests that LTH is modality specific, there are still some discussion points. The MRF lesion could have changed LTH specifically to acoustic stimuli. This is of importance since Jordan and Leaton tested specificity “only” from acoustically habituated rats to tactile stimulation.

In addition, in Jordan and Leaton (1982) the tactile stimulus had an acoustic component of 75 dB, which by itself did not elicit startle. Since the controls had lower startle amplitude to their complex tactile-acoustic stimulus, there might have been LTH in the controls to the acoustic component. If so, there might have been a general response amplitude difference between their tactile and their acoustic stimulation. As Jordan and Poore (1998) argue, new stimuli eliciting higher startle amplitudes are not easy to interpret in the light of LTH. When Jordan and Poore (1998) discussed the switch of acoustic quality in Jordan and Leaton (1982), they state critically: “However, this study did not attempt to equate stimulus intensities, and no animals were habituated to the noise stimulus and then switched to the pure tone.” In this sense, we wanted to look again into modality specificity, with tactile stimuli without acoustic artifact (using a silencer, Pilz et al., 2004), with stimuli roughly equaling stimulus intensities (the intensities of acoustic and tactile stimulation produced crossing LTH curves), and with animals trained to both stimulus modalities.

Jordan and Poore (1998) found no frequency specificity of LTH. LTH was stable if the stimulus frequency was changed from 10 to 22 kHz or vice versa, and if the same was done for 10 and 35 kHz. We thus know there is maximal generalization within this modality (Jordan and Poore, 1998), while there seems to be no generalization over modalities (Jordan and Leaton, 1982). Since our criticism of Jordan and Leaton (1982) is farfetched, our expectation was to confirm their result of stimulus specificity of LTH. If so, our results would extend this feature of LTH to mice, to unlesioned controls, and to cross-habituation in both directions. For this purpose, groups of mice were long-term habituated to either tactile or acoustic stimuli over a period of 9 days (with control groups for the respective time and background noise condition in the startle apparatus). On the 10th day they were tested using the different modality.

### Methods

#### Subjects

Subjects were 16 female and 28 male naïve Bl6 mice. They were divided into four groups. In both control groups there were 4 female and 6 male mice. There were 4 female and 8 male mice in the groups exposed to stimuli during training. Age, keeping, supplier, etc. were the same as in experiment 1.

#### Apparatus

The same apparatus and measures were used for acoustic stimulation as in Experiment 1. The only difference was the changed test cage (see below).

The apparatus for tactile stimulation is described and discussed in detail in Pilz et al. (2004); changes are described in the following. Tactile stimuli were airpuffs of 100 Pascal, measured at the center of the cage; the air pressure before the air valve solenoid was 2 bar = 29 psi. The airpuffs were delivered through a PVC tube centered on the side of the test cage, 7.5 cm from the center of the cage. The end of the round tube was compressed to an inner ellipsoid diameter of 1.2 × 0.25 cm. The tube was directed toward a cage with the same size as in experiment 1, with the single difference that it was elevated above the measuring platform by 3.5 cm by means of four stilts. The tube nozzle was at the same height, but directed to the middle of the cage; thus the air was directed slightly away from the measuring platform, which minimized the risk that tactile artifacts could be falsely measured as startle responses.

The airpuff characteristics were measured using a 1-inch (2.54-cm) microphone (Model 4145; Bruel and Kjaer, Naerum, Denmark). The airpuffs had a duration of 20 ms plus rise and decay times each lasting 8 ms and a pressure of 100 Pa. To reduce noise generated by the air valve solenoid, the air passed through a “silencer.” The silencer was a glass cylinder (diameter 5.3 cm, height 8.2 cm, volume 168 cubic cm) sealed with a rubber stopper. The rubber stopper had two holes, one for air inlet and the other for air outlet. The cylinder contained sound absorbing rubber foam (thickness 4.3 cm, on the side opposite the rubber stopper). To mask the sound of the airpuff itself, which had mainly low frequency components (Pilz et al., 2004), we performed all testing of the tactile startle response with background noise containing frequencies between 250 Hz and 20 kHz, with maximum intensity at 2 kHz. The noise was produced by a digital signal processing controlled system (Elf-Board with Siggen software; Medav, Uttenreuth, Germany), amplified and emitted by a second loudspeaker (Craaft HT 1640; Solton Music, Pocking, Germany) inside the sound-absorbing chamber. At a background noise level of 93 dB SPL RMS, the acoustic artifact of the tactile stimuli was completely masked.

Acoustic (14 kHz, 105 dB SPL, see Experiment 1) and tactile stimuli for all experiments had an intertrial interval of 15 s.

#### Procedure

As in Experiment 1, mice were adapted for 5 min daily to the startle apparatus. During each daily session the background noise was constant: 45 dB for the acoustic startle measures and 93 dB for the tactile startle measures. One-hundred stimuli were then given.

The four groups were:

Group acoustic: 9 days acoustic stimulation, then 1 day tactile stimulation.

Group acoustic-control: 9 days 45 dB background noise without stimulation, then 1 day with tactile stimulation.

Group tactile: 9 days tactile stimuli, then 1 day with acoustic stimuli.

Group tactile-control: 9 days 93 dB background noise without stimulation, then 1 day with acoustic stimuli.

#### Statistical analysis

Statistics were the same as in Experiment 1, with the following exceptions: since Mauchly's test was not significant, the uncorrected *F*-tests of the repeated measures ANOVA are reported for LTH (day and gender were statistically tested by two-factor ANOVAs).

### Results

**Figure 3:**
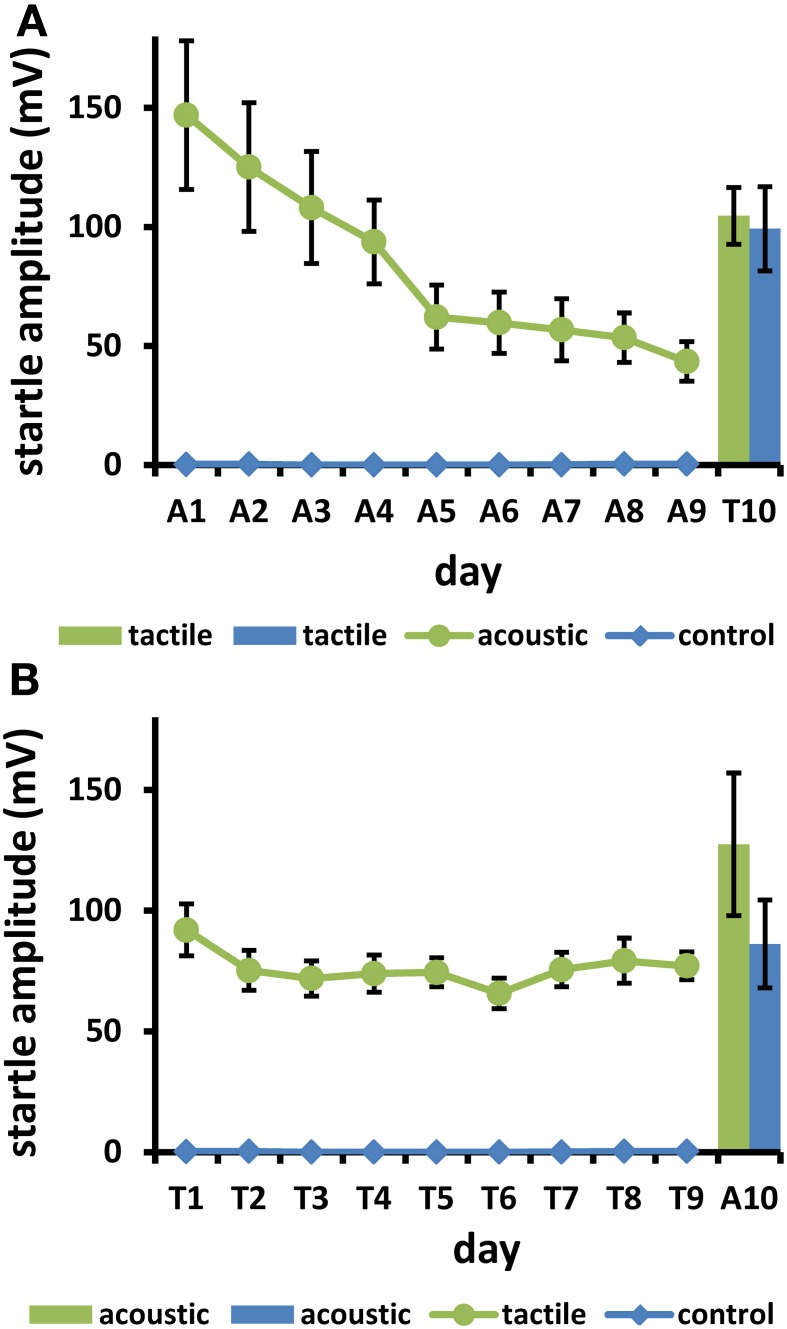
**(A)** Mice were startled with tactile stimuli on day 10 (T10) after 9 days (A1–9) of either 100 acoustic startle stimuli daily (group “acoustic”), or 9 days without acoustic stimulation (“control”). **(B)** Mice were startled on day 10 (A10) with acoustic startle stimuli, after 9 days (T1–9) with either 100 daily tactile startle stimuli (“tactile”) or without stimulation (“control”). The amplitude decrease during the first 9 days was significant in both stimulated groups (*p* < 0.05). There was no significant difference between the previously stimulated and the unstimulated groups on day 10 (*p* > 0.1. Bars: s.e.m.; *n* = 44).

The mice displayed strong LTH to acoustic stimuli on 9 days (mean day 9 = 29.6% of day 1; Figure 3A). The startle response to tactile stimulus on test day 10 was even slightly higher in the acoustic (i.e., LTH) group, compared to the acoustic-control group. There was no statistical difference between pretreatment groups. A two-factor ANOVA revealed a trend for a gender effect [*F*_(1, 18)_ = 3.81, *p* = 0.067], but no interaction [*F*_(1, 18)_ = 1.53, *p* = 0.23]. Most importantly, there was no significant effect of prestimulation on the test day [*F*_(1, 18)_ < 1]. Pairwise comparisons showed that tactile startle on day T10 (acoustic and acoustic control groups, Figure 3A) was not different from day T1 (tactile group, Figure 3B; uncorrected *t*-tests, *t* ≤ 1.1, *p* ≥ 0.27), but differed at least partly from day T9 [acoustic control: *t*_(20)_ = 1.9, *p* = 0.073; acoustically stimulated group: *t*_(22)_ = 2.8, *p* = 0.010].

There was only a weak LTH to tactile stimuli [mean day 9 = 83.8% of day 1; Figure 3B; *F*_(8, 80)_ = 2.33, *p* = 0.026; gender: *F* < 1; interaction gender × day: *F*_(8, 80)_ = 1.18, *p* = 0.32]. Mean responses on test day 10 to acoustic stimuli were even slightly higher in the tactile (LTH) group compared to the tactile-control group. The two-factor ANOVA yielded no effect of gender [gender, interaction gender × prestimulation: *F*_(1,18)_ < 1.8, *p* > 0.20], and no effect of prestimulation [*F*_(1, 18)_ = 2.32, *p* = 0.15]. Pairwise comparisons showed that acoustic startle on day A10 (tactile and tactile control groups, Figure 3B) was not different from day A1 (acoustic group, Figure 3A; uncorrected *t*-tests, *t* = 1.59, *p* = 0.13), but differed from day A9 (*t* = 2.51, *p* = 0.020).

### Discussion

#### No cross-habituation of LTH

There was clearly no cross-habituation if LTH was elicited by one stimulus modality, and then startle was tested by another stimulus modality. For acoustic LTH, i.e., 9 days of daily acoustic stimulation, this was very convincing since there was a large LTH to this modality of about 70% response decrease. If cross-habituation had occurred, we would expect a lower startle response to tactile stimuli after the acoustic LTH. However, the tactile response was the same in mice with acoustic LTH as in mice without acoustic training.

Tactile LTH training of 9 days produced a response decrease of only 16%. It is therefore not particularly convincing that no cross-habituation occurred. Nevertheless, the mice tested after this procedure did not startle less when subjected to acoustic stimuli than mice without tactile training.

Our results partially confirmed those of Jordan and Leaton (1982). They found LTH to acoustic stimuli to be modality specific in rats. There daily acoustic stimuli elicited more LTH in controls than in rats with a lesion of the MRF. This difference between lesioned and control rats was not extended to tactile stimulation. Since the MRF-lesion could have changed LTH specifically to acoustic stimuli, our results extend their finding not only to mice, but also to unlesioned controls.

While the decrease to tactile stimuli here was only 16%, in Plappert and Pilz (2005) it was 34% under comparable parameters. Borszcz et al. (1989) showed long-term sensitization counteracting LTH. Stimulus intensity for tactile stimuli was slightly higher than in the study of Plappert and Pilz (2005). This may have produced higher sensitization, and therefore smaller LTH. Absolute amplitudes elicited by acoustic and tactile stimuli were roughly the same (if averaged over all days; Figure 3). Hence, if our tactile stimuli induced sensitization, this should be specific for this modality, since it cannot be due to higher startle responses elicited by this stimulus. (Indeed, on day 1 tactile startle was lower than acoustic startle). Because in rats LTH to tactile stimuli is also relatively small compared to LTH to tonal stimuli (Jordan and Leaton, 1982), this might be characteristic for this modality.

However, another type of sensitization could have influenced the results. During acoustic stimulation days, the background noise SPL was 45 dB, while on days with tactile stimulation it was 93 dB. The 93 dB background noise was necessary to completely mask the acoustic artifacts produced by the airpuffs used for tactile stimulation, and it might have sensitized the mice. For this reason each experimental group had a control group with precisely the same background noise experience on the same days. E.g. the group which was subjected to acoustic stimuli (and thus 45 dB background noise) on days 1–9 (Figure 3A) had a control group of mice subjected to the same noise on days 1–9. When on day 10 the two groups were subjected to tactile stimuli for the first time, they also heard the higher background noise SPL for the first time, which might have influenced the results. However, the tactile startle amplitudes on this day were not different from those of the mice with tactile stimulation on day 1 (Figure 3B, T1), but higher than that of the same mice on day 9 (Figure 3, T9). Furthermore, the results were identical for the other direction of cross-habituation. Thus, since the quantitative and statistical results are as expected if cross-habituation is absent, and since the amplitudes of tactile and acoustic startle from the beginning were different, and furthermore, since the LTH to acoustic and tactile startle were different, we find it unlikely that these exact results are due to background noise changes. We cannot rule out influences of the background noise differences on these results; however, we believe the results indicate absence of cross-modality in LTH.

Testing another change of stimulus characteristics, Jordan and Poore (1998) found no specificity for stimulus frequency. LTH remained constant if stimulus frequency was changed by one or two octaves. The lack of generalization was restricted to LTH of startle, but could be measured in other paradigms. We feel that Jordan and Poore correctly argue that response latency of startle is much shorter than in the other paradigms. Typically latency of startle in rodents can be as short as 7 ms (Plappert and Pilz, 2013), electromyographically even below 6 ms (Caeser et al., 1989). This is probably too short for frequency specific processing. Therefore, although stimulus generalization, one of the nine (Thompson and Spencer, 1966) respectively 10 (Rankin et al., 2009) key characteristics of habituation, is absent inside the auditory stimulus modality, it is demonstrable between stimulus modalities.

Although LTH was different on training days 1–9 to acoustic and tactile stimuli, in both cases the startle elicited afterwards by the different modality was never lower in the LTH-trained mice than in the controls. Thus, we conclude that LTH in mice is stimulus modality specific. Furthermore, because this confirms the conclusions found in literature on rats, we believe that this specificity applies to rodents in general.

#### Assumed neuronal action of LTH on startle pathway

As already pointed out above, the neuronal pathway of acoustic startle consists of primary and secondary auditory neurons projecting onto giant neurons in the PnC, which are the sensorimotor interface and project themselves onto motoneurons (Plappert and Pilz, 2013). Tactile input from the face (i.e., relayed by the sensory trigeminal nerve) also comes from secondary sensory neurons to the giant neurons (Li and Yeomans, 1999; Scott et al., 1999; Simons-Weidenmaier et al., 2006). Whether and how haptic input from the body is relayed to the giant neurons is unclear [discussed in Pilz et al. (2004)]. Current discussions assume that they are also connected to giant neurons (e.g. Simons-Weidenmaier et al., 2006).

STH of startle is modality specific (similar to LTH shown here). Startle decrease elicited by acoustic stimuli is not transferred to the tactile modality; neither is the startle decrease elicited by tactile stimuli transferred to the acoustic modality (Pilz et al., 2004; Vogel and Wagner, 2005; Ponce et al., 2011). Since the sensory inputs to the common PnC interface are distinct for auditory and haptic information, and since several publications propose that the pathway from PnC neurons onwards is identical for both sensory modalities (Li and Yeomans, 1999; Scott et al., 1999; Simons-Weidenmaier et al., 2006), STH must be situated in the respective sensory branches. Indeed, it has been shown that this process probably takes place in the synapse of secondary sensory neurons on giant PnC neurons (Simons-Weidenmaier et al., 2006).

Here we demonstrated that LTH is also stimulus modality specific. With the same logic as for STH, LTH also should act on the respective sensory branches of the startle pathway. We cannot exclude unknown additional motor branches for both sensory modalities studied, as found for vestibular startle (Li et al., 2001). However, since the current knowledge regarding startle circuitry does not (yet) include this possibility for acoustic and tactile startle, we are confident that LTH acts prior to the motor part of the pathway of this response system. In addition, after our findings in Experiment 1, this is a second indication for a sensory location of neuronal LTH action.

This neural implementation would restrict LTH generalization. As pointed out above, startle latency is probably too short for stimulus specificity inside one modality. However, by acting differentially on sensory branches, some specificity of LTH would be preserved, which would prevent unnecessary inhibition of modality-different stimulation.

## General discussion

### Startle LTH depends on stimulus type used

LTH differed for different types of stimuli. In both experiments there was a strong LTH to 14 kHz tonal startle stimuli. LTH was significant but much weaker regarding tactile stimuli and almost non-existent to noise stimuli in our pretest. As already discussed in Experiment 1, the tonal stimuli were exactly the same each time they were presented, and thus might be best suited to elicit LTH. Noise stimuli are not constant in course of amplitude or frequency, which might interfere with their ability to elicit LTH. Since mice move in the test cages, the airpuffs hit different parts of the body. Pilz et al. (2004) show that this movement did not influence STH in their study. Nevertheless, haptic stimulation of different parts of the skin may also prove to be an inconsistent stimulus with respect to LTH. However, Jordan and Poore (1998) found evidence that refutes this explanation, i.e., they found LTH to explicitly non-constant stimuli. In their experiments, LTH was independent of stimulus frequency during testing compared to training. Whatever the correct explanation, tonal stimuli, such as 14 kHz, which is in the best hearing range of mice (Ehret, 1983), seem to be most useful for LTH training.

### Startle LTH is a non-associative learning paradigm

The strongest suggestion that startle LTH is non-associative can be derived from experiment 1, showing that LTH is constant after context change, thereby confirming original literature (Marlin and Miller, 1981; Jordan et al., 2000). Since Jordan et al. (2000) also show that for other response systems LTH is associative, the non-associative nature of LTH shown here is restricted to the startle response system. If this process is non-associative, it must depend solely on stimulus characteristics. This obviously is the case, since LTH depends on the stimulus modality used (Experiment 2). If LTH was elicited by acoustic or tactile stimuli, and then stimulus modality was changed (to tactile or acoustic, respectively), there was no transfer of LTH from one to the other modality. Thus, taken together, all data suggest that startle LTH in mice is non-associative, as discussed previously for rats (Marlin and Miller, 1981; Leaton and Supple, 1986; Jordan and Poore, 1998; Jordan et al., 2000).

### Proposed neuronal action of LTH on startle pathway

Two outcomes suggest the same location of neural action of LTH on the startle pathway. In Experiment 1, LTH was shown to possibly have a strong diminishing effect not only on startle amplitude, but also on STH of startle (in the one strain exhibiting reliable STH); to date, STH has only been found in the sensory branches of the startle pathway (Simons-Weidenmaier et al., 2006). In the second experiment, LTH proved to be sensory modality specific. This also indicates that the modality specific neuronal input branches of the startle pathway are the location of LTH action.

### Summary

A context change did not disrupt startle LTH; neither was there a transfer of LTH from one stimulus modality to the other (tactile to acoustic or vice versa). So, similar to previous data from rats, our results indicate that LTH in mice is a non-associative stimulus modality specific learning paradigm. Results from both experiments together with data from the literature suggest that the neuronal action of LTH is a chronic inhibition aiming at the modality specific sensory input branches of the startle pathway. Moreover, we found the best LTH to tonal startle stimuli compared to noise pulses or tactile airpuffs.

## Authors contributions

Peter K. D. Pilz planned the experiments, tested data statistically and wrote the manuscript. Stephan W. Arnold and Anja T. Rischawy conducted the experiments, including optimization of the contexts and tactile stimulation, and compiled part of the descriptive statistics as well. Claudia F. Plappert planned experiments and contributed to the discussion and the manuscript. There was no conflict of interests for any author.

### Conflict of interest statement

The authors declare that the research was conducted in the absence of any commercial or financial relationships that could be construed as a potential conflict of interest.
